# Increased hospital treatment volume of splenic injury predicts higher rates of successful non-operative management and reduces hospital length of stay: a Swiss Trauma Registry analysis

**DOI:** 10.1007/s00068-020-01582-z

**Published:** 2021-01-23

**Authors:** Joël L. Lavanchy, Luciane Delafontaine, Tobias Haltmeier, Piotr Bednarski, Beat Schnüriger, the Swiss Trauma Registry

**Affiliations:** 1 Department of Visceral Surgery and Medicine, Acute Care Surgery Team, Inselspital, Bern University Hospital, University of Bern, 3010 Bern, Switzerland; 2 Department of Emergency Medicine, Inselspital, Bern University Hospital, University of Bern, Bern, Switzerland; 3 Swiss Trauma Board, Basel, Switzerland

**Keywords:** Splenic injury, Treatment volume, Non-operative management, Outcomes, Swiss trauma registry

## Abstract

**Purpose:**

First time analysis of the epidemiology, management and outcomes of patients with splenic injuries in Switzerland. This study aims to assess the effect of hospital treatment volume on successful non-operative management (NOM) in splenic injuries.

**Methods:**

A multicentric registry-based study including all patients with splenic injuries entered into the Swiss Trauma Registry from 2015 to 2018 was conducted. Patients were stratified according to the hospitals treatment volume of splenic injuries. Primary outcome was the rate of successful NOM.

**Results:**

During the 4-year study period, 652 patients with splenic injury were included in the study. Median age of the study population was 42 (IQR 27–59) years, and median ISS was 26 (20–34). The overall rate of successful NOM was 86.5%. Median HLOS was 13 (8–21) days. In-hospital mortality was 7.2% (*n = *47). The mean number of patients with splenic injuries per center and year was 14. Five out of 12 Level I trauma centers treating more patients than the mean (≥ 15/year) were defined as high-volume centers.

Multivariable analysis adjusting for differences in baseline and injury characteristics revealed treatment in a high-volume center as an independent predictor for successful NOM (OR 2.15, 95% CI 1.28–3.60, *p* = 0.004) and shorter HLOS (RC − 2.39, 95% CI − 4.91/− 0.48, *p* = 0.017), however, not for reduced in-hospital mortality (OR 0.92, 95% CI 0.39–2.18, *p* = 0.845).

**Conclusion:**

Higher hospital treatment volume was associated with a higher rate of NOM and shorter HLOS, but not lower mortality. These results constitute the basis for further quality improvement in the care of splenic injury patients within the trauma system in Switzerland.

## Introduction

In the past decades, the management of splenic injuries fundamentally changed. In hemodynamically stable patients, the incidence of splenectomies decreased significantly in favor of a non-operative, splenic preserving management (NOM) [1–3]. Currently, up to 90% of splenic injuries are treated non-operatively. Furthermore, the increasing use of angioembolization has resulted in lower rates of failed NOM [4, 5]. However, this requires an interventional around-the-clock-service. Splenic preservation is feasible in most circumstances and has been shown to reduce mortality and short- and long-term morbidity in patients suffering from severe splenic trauma [6–8].

Over 15 years ago, multicenter studies in the USA showed that treatment volume is associated with reduced hospital length of stay (HLOS) and mortality in trauma care [9, 10]. However, regarding the volume–outcome relationship, no literature from Europe and in particular for patients with splenic injury is available.

Since the year 2015, the Swiss Trauma Registry (STR), the first and only national trauma database in Switzerland, is operative [11]. Twelve academic and non-academic teaching hospitals were certified in 2011 as Level I trauma centers and were obligated to participate in the STR.

The current study aimed to assess for the first time the epidemiology, management and outcomes of patients with splenic injuries in Switzerland. We hypothesize that higher trauma center treatment volume improves the rate of successful NOM in patients with splenic injuries.

## Methods

### Study design and outcome measures

This is a multicentric registry-based observational cohort study. Patients entered into STR from 01/01/2015 to 31/12/2018 were screened for inclusion. Inclusion criteria were splenic injury and initial treatment in Switzerland. Primary outcome measure was the rate of successful NOM. Secondary outcomes comprised HLOS and in-hospital mortality.

### Data acquisition and definitions

Data were obtained from all 12 Level I trauma centers in Switzerland through the STR. Number and severity of splenic injuries were identified using Abbreviated Injury Scale (AIS) version 2005 (update 2008) abdomen codes (544,212.2, 544,214.3, 544,222.2, 544,224.3, 544,226.4, 544,228.5) [12]. Grade I and II splenic injuries were defined as low grade, grade III splenic injuries as intermediate grade and grade IV and V splenic injuries as high grade. Imaging studies at admission were conducted according to Advanced Trauma Life Support 9^th^ edition guidelines [13]. Angioembolization and operative treatment of splenic injuries were identified using Swiss standardized operation (CHOP) codes (39.79.25/26/29/35/36/45/46/55/56/64–66). Initial non-operative treatment with or without angioembolization was defined as attempted NOM. In accordance with the literature, all splenectomies (CHOP 41.43, 41.5) or spleen-preserving operations for hemostasis [suture repair, splenorrhaphy (CHOP 41.95.10, 41.95.99, 41.99)] later than 8 h after admission were considered as failed NOM [14]. Successful angioembolization after 8 h or successful re-angioembolization was defined as successful NOM. The twelve certified Swiss trauma centers were stratified according to treatment volume of splenic injuries. The mean number of patients with splenic injuries per center and year was 14. Five centers treated more patients than the mean (≥ 15/year) and were defined as high-volume centers. The remaining 7 centers treated < 15 patients/year and were defined as low-volume centers.

### Statistical analysis

Values were reported as numbers and percentages, means or medians and interquartile range (IQR), as appropriate. Normality of distribution was assessed using the Shapiro–Wilk test. Categorical variables were compared using Fisher’s exact test and continuous variables using Mann–Whitney *U* test. The effect of treatment volume on the rate of successful NOM and secondary outcomes was adjusted in multivariable analysis. Patient and injury characteristics (age, gender, Glasgow Coma Scale (GCS) score at admission, injury severity score (ISS), AIS, severity of splenic injury) and vital signs at admission (arterial blood pressure, heart rate) were assessed in univariable analysis and included into the multivariable model if the *p* value was < 0.2. Linear or logistic regression analysis was used for continuous or binary outcomes, respectively. Results were reported as standardized regression coefficients (RC) or odds ratios (OR) with 95% confidence intervals (CI). *p* values ≤ 0.05 were considered statistically significant. Statistical analyses were performed using SPSS statistics version 25 (IBM Corporation, Armonk, New York). Figures were created using Matplotlib for Python [15].

### Ethical requirements

The STR fulfills the requirements of the Swiss Human Research Act and has been registered as a multicentric registry by the cantonal ethics committee of Bern (2014-00,296). The study protocol was approved by the Swiss Trauma Board and the cantonal ethics committee of Bern, Switzerland (201-00,647). This study is reported in accordance with the STROBE (Strengthening the Reporting of Observational Studies in Epidemiology) statement [16].

## Results

**Fig. 1:**
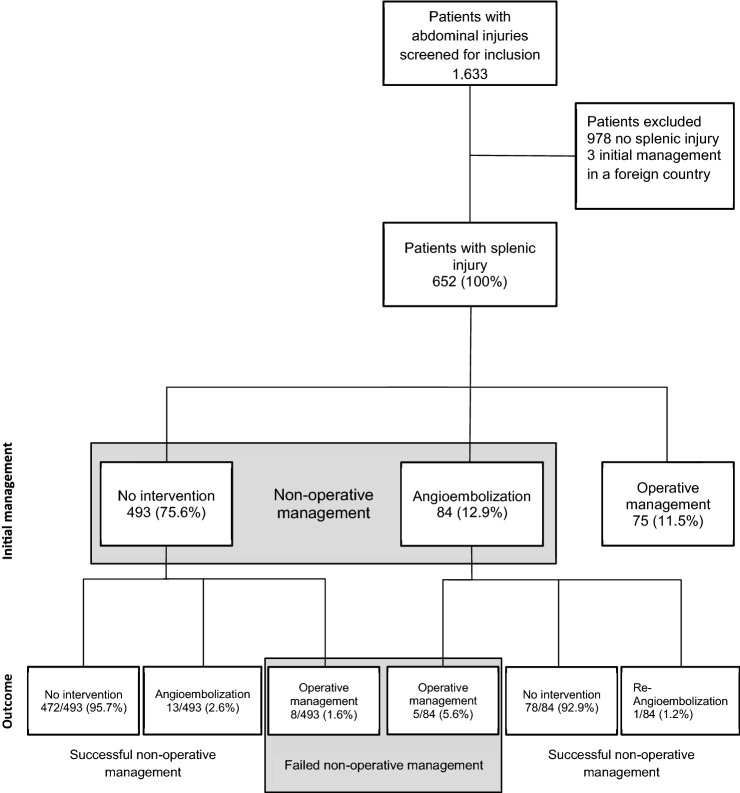
Study outline

From 2015 to 2018, 11,440 trauma patients admitted to one of the 12 participating centers were enrolled in the STR. Thereof, 1,633 patients (14.3%) with abdominal trauma were screened for inclusion. A total of 652 patients treated for splenic injury were identified and included into the analysis (Fig. 1).

Median (IQR) age of the study population was 42 (27–59) years and median ISS 26 (20–34). Injury mechanism was blunt in 601 patients (92.2%) and penetrating in 51 patients (7.8%). In total, 134 patients (20.6%) were referred from non-Level I hospitals. Overall, 74.8% (*n = *488) underwent computer tomography and 57.2% (*n = *373) underwent sonography (focused assessment with sonography for trauma, FAST) at hospital admission. Low-grade injuries accounted for 48.0% (*n = *308), intermediate-grade injuries for 23.4% (*n = *150) and high-grade injuries for 28.7% (*n = *184). Patient’s baseline characteristics are displayed in Table 1.

**Table 1:** Baseline characteristics of patients with splenic injury stratified by treatment volume of centers

	Overall (*N = *652)	High-volume centers (*n = *397)	Low-volume centers (*n = *255)	*p* value
Age, years, median (IQR)	42 (27–59)	42 (26–58)	44 (29–60)	0.147^a^
Male gender, *n* (%)	483 (74.1)	299 (75.3)	184 (72.2)	0.410^b^
GCS, *n* (%)				
3–8	127 (20.3)	77 (20.4)	50 (20.2)	1.000^b^
9–12	35 (5.6)	18 (4.8)	17 (6.9)	0.289^b^
13–15	463 (74.1)	282 (74.8)	181 (73.0)	0.641^b^
ISS, median (IQR)	26 (20–34)	29 (20–36)	24 (28–29)	< *0.001*^*a*^
AIS ≥ 4, *n* (%)				
Head	76 (11.7)	65 (16.4)	11 (4.3)	< *0.001*^*b*^
Chest	152 (23.3)	106 (26.7)	46 (18.0)	*0.011*^*b*^
Abdomen	245 (37.6)	169 (42.6)	76 (29.8)	*0.001*^*b*^
Extremities	73 (11.2)	57 (14.4)	16 (6.3)	*0.001*^*b*^
Severity of splenic injury, *n* (%)				
Low (grade I & II)	308 (48.0)	194 (48.9)	114 (46.5)	0.423
Intermediate (grade III)	150 (23.4)	86 (21.7)	64 (26.1)	
High (grade IV & V)	184 (28.7)	117 (29.5)	67 (27.3)	
Vital signs at admission, *n* (%)				
Syst. blood pressure ≤ 100 mmHg	155 (23.9)	91 (23.1)	64 (25.1)	0.573^b^
Heart rate ≥ 100 bpm	250 (38.5)	155 (39.3)	95 (37.3)	0.621^b^
Respiration rate ≥ 22 per min	95 (24.9)	58 (25.1)	37 (24.5)	1.000^b^
Oxygen saturation ≤ 90%	68 (12.7)	36 (10.9)	32 (15.7)	0.110^b^
Blood analysis, *n* (%)				
Hemoglobin ≤ 80 g/L	47 (7.2)	31 (10.5)	16 (6.3)	0.092^b^
Thrombocytes ≤ 50 G/L	3 (0.6)	2 (0.7)	1 (0.4)	1.000^b^
Lactate > 2.0 mmol/L	224 (34.4)	111 (28.0)	113 (44.3)	0.075^b^
INR > 1.2	112 (20.6)	69 (23.5)	43 (17.1)	0.071^b^

*NOM* non-operative management, *IQR* interquartile range, *GCS* Glasgow Coma Scale, *ISS* injury severity score, *AIS* abbreviated injury scale, *INR* international normalized ratio

Statistical significant *p* values are italic

^a^Mann–Whitney *U* test, ^b^Fisher’s exact test

The overall attempted NOM rate was 88.5% (*n = *577). Angioembolization was applied as primary treatment in 84 patients (12.9%). There were 13 patients (2.0%) that underwent splenic operation later than 8 h after admission and were defined as failed NOM. The in-hospital mortality rate was 7.2% (*n = *47). Of those 47 patients that died within the hospital, 51.1% (*n = *24) had severe traumatic brain injury (AIS head ≥ 4), and 38.3% (*n = *18) had severe chest injuries (AIS chest ≥ 4). There was no fatality in the group of 13 patients that failed NOM.

**Fig. 2:**
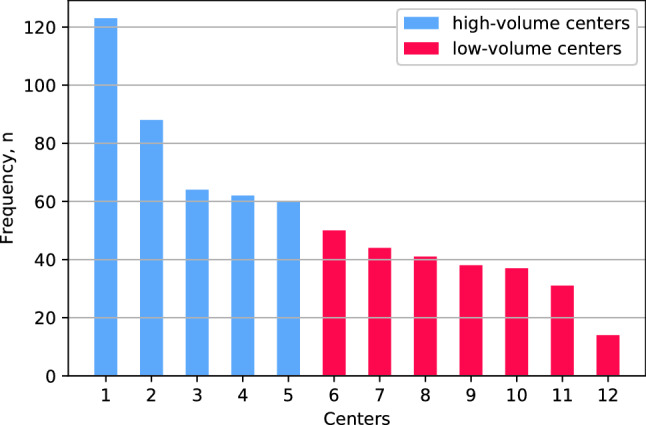
Numbers of blunt splenic injuries treated per center 2015–2018

The mean number of splenic injuries per center during the 4-year study period was 54 with a range of 14–123 (Fig. 2). Of the study population, 60.9% (*n = *397) were treated in high-volume centers (≥ 15 splenic injuries per year), and 39.1% (*n = *255) were treated in low-volume centers (< 15 splenic injuries per year).

Patients treated in high-volume centers had significantly higher median (IQR) ISS compared to patients treated in low-volume centers (29 (20–36) vs 24 (28–29), *p* < 0.001). The proportion of patients with AIS head ≥ 4 (16.4% vs. 4.3%, *p* < 0.001), AIS chest ≥ 4 (26.7% vs. 18.0%, *p* = 0.011), AIS abdomen ≥ 4 (42.6% vs. 29.8%, *p* = 0.001) and AIS extremities ≥ 4 (14.4% vs. 6.3%, *p* = 0.001) was significantly higher in high-volume centers.

**Fig. 3:**
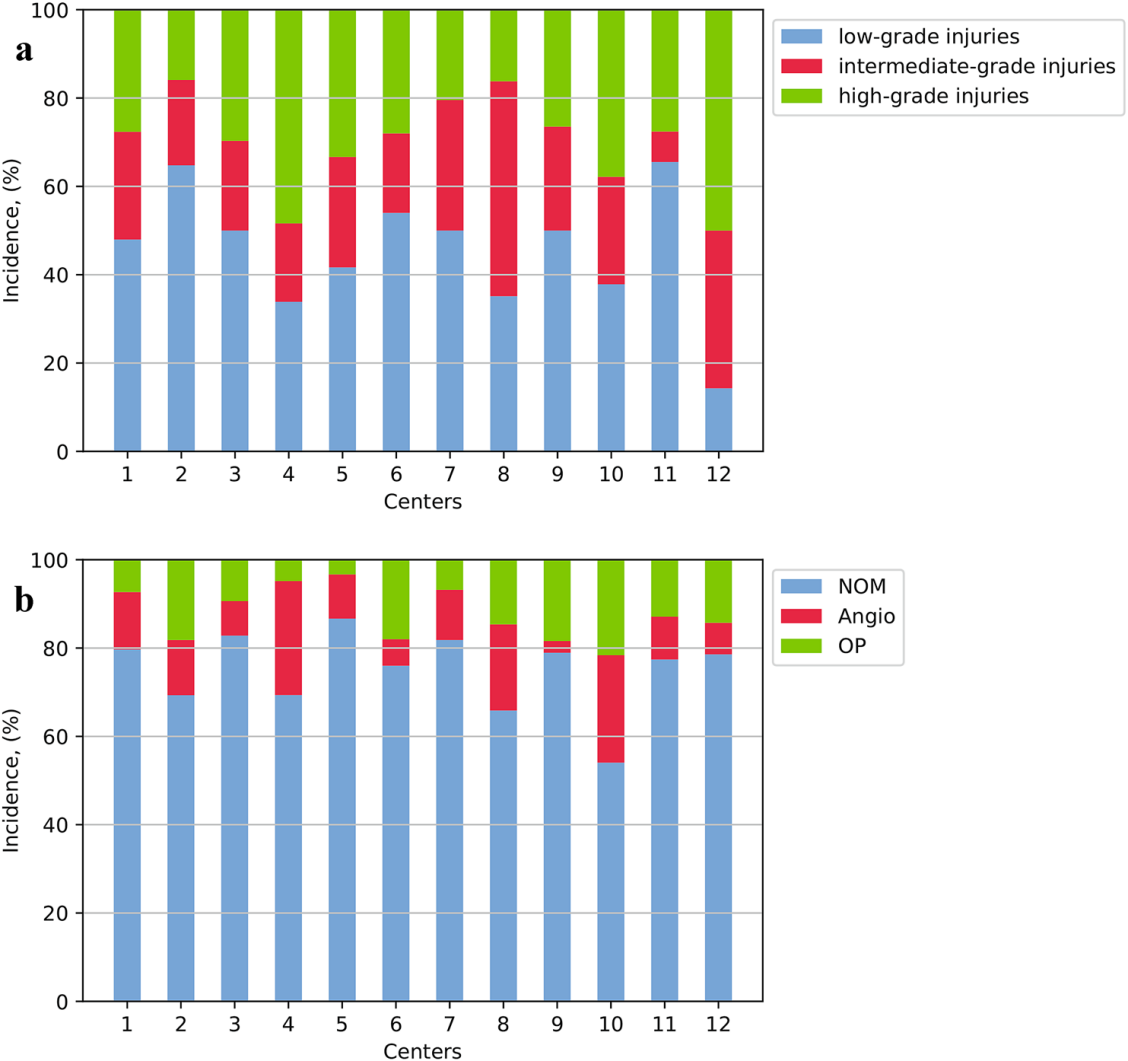
**a** Severity of splenic injuries stratified by center, **b** management of splenic injuries stratified by center (OP: operative, angio: angioembolization, NOM: non-operative management)

The proportion of intermediate- and high-grade splenic injuries varied considerably across centers (Fig. 3a). However, when comparing high- versus low-volume centers, the severity of splenic injury was not statistically different (Table 1).

**Fig. 4:**
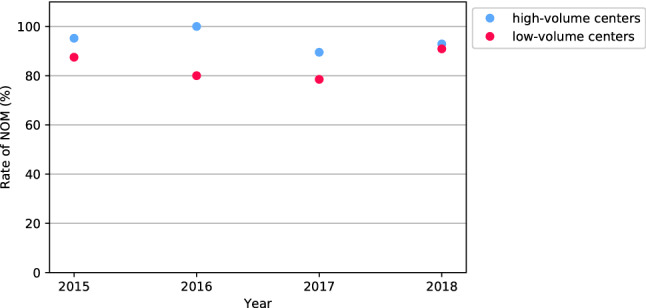
Median rate of attempted non-operative management (NOM) of splenic injuries over time stratified by treatment volume of centers

The proportion of patients with splenic injuries with successful NOM was significantly higher in patients treated at high- compared to low-volume centers (88.9% vs. 82.7%, *p* = 0.026) (Table 2). The rate of primary angioembolized patients was not significantly different between high- versus low-volume centers (13.6% vs. 11.8%, *p* = 0.550). Primary operative treatment was significantly more frequent in low- compared to high-volume centers (15.3% vs. 9.1%, *p* = 0.017). There was a trend toward more spleen-preserving surgical procedures in low- versus high-volume centers (4.7% vs. 2.0%, *p* = 0.063) (Table 2). Figure 4 shows the rates of attempted NOM during the four-year study period. There was a trend toward increased NOM in low-volume centers.

**Table 2:** Outcomes of patients with splenic injury stratified by treatment volume of centers

	High-volume centers (*n = *397)	Low-volume centers (*n = *255)	*p* value
Outcome, *n* (%)			
Successful NOM	353 (88.9)	211 (82.7)	*0.026*^*a*^
Primary operative management	36 (9.1)	39 (15.3)	*0.017*^*a*^
Failed NOM	8 (2.0)	5 (2.0)	1.000^a^
Splenectomy	36 (9.1)	32 (12.5)	0.189^a^
Spleen-preserving surgery	8 (2.0)	12 (4.7)	0.063^a^
Overall splenic preservation	361 (90.9)	223 (87.5)	0.189

*NOM* non-operative management

Statistical significant *p* values are italic

^a^Fisher’s exact test

Multivariable regression analysis revealed treatment in a high-volume center (OR 2.15, 95% CI 1.28–3.60, *p* = 0.004) as an independent predictor for successful NOM. GCS ≤ 8 (OR 0.51, 95% CI 0.27–0.96, *p* = 0.036), ISS ≥ 25 (OR 0.52, 95% CI 0.29–0.97, *p* = 0.041), high-grade injury (OR 0.13, 95% CI 0.07–0.22, *p* < 0.001) and systolic blood pressure ≤ 100 mmHg (OR 0.52, 95% CI 0.30–0.91, *p* = 0.021) were independent factors ruling against successful NOM.

Shorter HLOS was independently predicted by treatment in a high-volume center (RC − 2.39, 95% CI − 4.91/− 0.48, *p* = 0.017) and high-grade injury (RC − 2.61, 95% CI − 5.81/− 0.82, *p* = 0.009), whereas longer HLOS was independently predicted by ISS ≥ 25 (RC 2.47, 95% CI 0.64–5.61, *p* = 0.014), AIS extremities ≥ 4 (RC 3.38, 95% CI 2.61–9.85, *p* = 0.001), systolic blood pressure ≤ 100 mmHg (RC 2.87, 95% CI 1.25–6.68, *p* = 0.004) and heart rate ≥ 100 bpm (RC 2.17, 95% CI 0.24–4.84, *p* = 0.031).

In-hospital mortality was independently predicted by higher age (OR 1.07, 95% CI 1.04–1.09, *p* < 0.001), GCS ≤ 8 (OR 19.88, 95%CI 7.47–52.88, *p* < 0.001) and AIS head ≥ 4 (OR 3.39, 95%CI 1.33–8.63, *p* = 0.010) (Table 3). No difference in mortality was found when comparing high- with low-volume center.

**Table 3:** Uni- and multivariable regression analysis

	Univariable		Multivariable	
	RC/OR (95% CI)	*p* value	RC/OR (95% CI)	*p* value
Successful NOM				
High-volume center	1.67 (1.07–2.63)	*0.025*	2.15 (1.28–3.60)	*0.004*
GCS 3–8	0.50 (0.30–0.82)	*0.007*	0.51 (0.27–0.96)	*0.036*
ISS ≥ 25	0.33 (0.20–0.56)	< *0.001*	0.52 (0.29–0.97)	*0.041*
AIS thorax ≥ 4	0.69 (0.42–1.13)	0.139	0.86 (0.46–1.60)	0.628
High-grade splenic injury	0.14 (0.09–0.24)	< *0.001*	0.13 (0.07–0.22)	< *0.001*
Syst. blood pressure ≤ 100 mmHg	0.33 (0.21–0.52)	< *0.001*	0.52 (0.30–0.91)	*0.021*
Heart rate ≥ 100 bpm	0.47 (0.30–0.74)	< *0.001*	0.60 (0.35–1.01)	0.055
HLOS				
High-volume center	− 1.61 (− 4.10–0.41)	0.108	− 2.39 (− 4.91/− 0.48)	*0.017*
GCS 3–8	4.08 (3.01–8.59)	< *0.001*	1.22 (− 1.08–4.65)	0.222
ISS ≥ 25	4.96 (3.32–7.67)	< *0.001*	2.47 (0.64–5.61)	*0.014*
AIS thorax ≥ 4	3.53 (2.06–7.22)	< *0.001*	1.15 (− 1.13–4.36)	0.249
AIS extremities ≥ 4	5.40 (5.98–12.83)	< *0.001*	3.38 (2.61–9.85)	*0.001*
High-grade splenic injury	− 2.96 (− 6.11− 1.24)	*0.003*	− 2.61 (− 5.81/− 0.82)	*0.009*
Syst. blood pressure ≤ 100 mmHg	5.24 (4.24–9.32)	< *0.001*	2.87 (1.25–6.68)	*0.004*
Heart rate ≥ 100 bpm	4.32 (2.69–7.18)	< *0.001*	2.17 (0.24–4.84)	*0.031*
Mortality				
High-volume center	1.56 (0.82–2.98)	0.177	0.92 (0.39–2.18)	0.845
Age	1.03 (1.02–1.04)	< *0.001*	1.07 (1.04–1.09)	< *0.001*
GCS 3–8	19.07 (8.84–41.17)	< *0.001*	19.88 (7.47–52.88)	< *0.001*
ISS ≥ 25	7.73 (3.02–19.81)	< *0.001*	3.07 (0.93–10.15)	0.066
AIS head ≥ 4	11.10 (5.86–21.02)	< *0.001*	3.39 (1.33–8.63)	*0.010*
AIS thorax ≥ 4	2.18 (1.18–4.05)	*0.013*	1.25 (0.53–2.96)	0.615
Syst. blood pressure ≤ 100 mmHg	3.57 (1.94–6.56)	< *0.001*	1.25 (0.54–2.91)	0.604
Heart rate ≥ 100 bpm	2.52 (1.38–4.62)	*0.003*	1.45 (0.63–3.30)	0.381

*NOM* non-operative management, *GCS* Glasgow Coma Scale, *ISS* injury severity score, *AIS* abbreviated injury scale, *HLOS* hospital length of stay, *RC* regression coefficient, *OR* odds ratio, *CI* confidence interval

Statistical significant *p* values are italic

## Discussion

This multicentric registry-based analysis aimed to determine the effect of treatment volume on outcomes in the management of splenic injuries. Overall, 652 patients with splenic injuries were treated in the 12 trauma centers in Switzerland, resulting in a mean of 14 cases/year/center. There were 6 centers defined as high-volume (> 15 cases/year) that managed 60% of the entire population. Treatment in a high-volume center revealed to be an independent predictor for successful NOM and shorter HLOS, however, without impact on in-hospital mortality.

This study revealed a 5.7% (652/11,440) prevalence of splenic injury in patients with an ISS > 15 or AIS head > 2 entered into the STR from 2015 to 2018. A Scottish registry-based analysis showed a 1.27% (672/52,887) prevalence among polytraumatized patients of splenic injury during an 11-year period [17]. However, inclusion criteria of the Scottish trauma registry (age > 13 years and hospitalization ≥ 3 days or fatal trauma or inter-hospital transfer) were more liberal.

In the current study, there was a considerable inter-hospital variability in the severity of splenic injuries. Nevertheless, comparing the entire Swiss splenic injury patients to a multi-center study from the USA, the proportion of high-grade injuries was similar (28.7% vs. 24.8%) [18].

The current study showed an 87% successful NOM rate. This was considerably higher than the 56–73% NOM rates reported in two US and a Taiwanese registry-based analyses despite comparable injury characteristics (ISS and grade of splenic injury) to the current study [18–20]. A dense network of trauma centers—twelve Level I centers serving a population of 8.5 million inhabitants—and the high rate of angioembolization (14.8% in the current study vs. 8.8% in a multicenter US study [4]) might contribute to this finding.

This study demonstrates a higher rate of successful NOM in high- compared to low-volume centers despite higher ISS and more severe injury characteristics. However, operative management included also spleen-preserving surgeries resulting in a similar rate of splenic preservation when comparing high- and low-volume centers.

To our knowledge, there is only one previous study assessing the effect of treatment volume on management and outcomes of splenic injuries in adults [19]. This analysis of the State of Pennsylvania’s trauma database used American College of Surgeons recommendations on optimal trauma center volume for Level I designation as criterion to stratify into high-volume and low-volume center. However, the actual number of splenic injuries treated per center was not provided. Nevertheless, similar to the current study, patients with splenic injuries admitted to low-volume centers were more likely to be treated operatively. Furthermore, as in the current study mortality was not different between high- and low-volume centers.

The results of this study are limited to the analysis of the STR. Trauma facilities not accredited by the Swiss Trauma Board are not included in the STR. Therefore, the generalizability of our findings is limited to Level I trauma centers in Switzerland. A failure of an attempted NOM was not documented within the STR; therefore, as described in the literature, a failure of the NOM was defined as undergoing spleen-related surgery ≥ 8 h after admission [14]. Although quality monitoring by external audit there might be interobserver variability regarding grading of injury characteristics between the participating centers.

In conclusion, the care of patients with splenic injuries in the twelve Swiss Level I trauma centers is of high quality and comparable to international standards. Efforts to improve centralization of patients with splenic injuries to one of these trauma centers are advocated as higher hospital treatment volume was associated with a higher successful NOM rate and shorter HLOS. These results constitute the basis for further quality improvement in the care of splenic injury patients within the trauma system in Switzerland.

## Data Availability

The data of this project are available upon request.
